# Virtualization as a New Scaling Law for Semiconductor Devices Beyond Geometric Scaling

**DOI:** 10.1002/smll.202510426

**Published:** 2026-02-26

**Authors:** Zeheng Wang, Xinghuan Chen, Fanfan Lin, Xinze Li, Fangzhou Wang, Songnan Guo, Simin Yu, Liang Li, Jing‐Kai Huang

**Affiliations:** ^1^ Manufacturing CSIRO Sydney NSW Australia; ^2^ China Electronic Product Reliability and Environmental Testing Research Institute Guangzhou China; ^3^ Zhejiang University – University of Illinois Urbana‐Champaign Institute Zhejiang China; ^4^ University of Arkansas Fayetteville Arkansas USA; ^5^ Shenzhen Institute for Advanced Study University of Electronic Science and Technology of China Shenzhen China; ^6^ NVIDIA Semiconductor Technology (Shanghai) Co., Ltd Shanghai China; ^7^ Intel Chengdu Products Co., Ltd Chengdu China; ^8^ Academy for Advanced Interdisciplinary Studies Peking University Beijing China; ^9^ School of Materials Science and Engineering University of New South Wales Sydney NSW Australia

**Keywords:** artificial intelligence, fabrication, machine learning, scaling law, semiconductor devices

## Abstract

As Moore's‐law–driven geometric scaling nears physical, economic, and sustainability limits, semiconductor progress is increasingly constrained by the cost and latency of physical iteration. This Perspective argues that AI enables virtualization as a complementary scaling law: progress scales with how much trustworthy virtual evidence can replace exhaustive fabrication, testing, and qualification across the device lifecycle. We show how virtualization emerges in i) design and modeling via surrogate and physics‐informed learning, inverse design, and uncertainty‐aware exploration; ii) fabrication and packaging via digital twins, virtual metrology, and reinforcement learning; and iii) qualification via defect inference and reliability modeling that provide earlier risk signals. We outline boundary conditions—trust and uncertainty, cross‐stage coherence, sustainability, and governance—and argue that future innovation will depend not only on geometric shrinkage, but also on the fidelity, integration, and stewardship of virtual evidence.

## Graphical abstract

AI‐enabled semiconductor scaling law. Virtualization emerges as an AI‐enabled scaling law for semiconductors, where progress depends on replacing physical iteration with credible virtual evidence. Surrogate modeling accelerates design‐space exploration, digital twins virtualize process learning, and defect‐to‐reliability inference advances qualification. The limiting factors move from feature length to evidence quality: validation, uncertainty, lifecycle coherence, and governance set the achievable scaling rate.

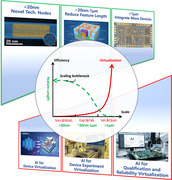

**Abbreviations**
Abbrev.: Full term
A2C: Advantage Actor–Critic
AI: Artificial Intelligence
AMADAP: Autonomous Material and Device Acceleration Platform
ANN: Artificial Neural Networks
CDLDM: Cross Domain Latent Diffusion Model
CML: Classical Machine Learning
CMP: Chemical Mechanical Planarization
CNN: Convolutional Neural Network
DAP: Device Acceleration Platform
DDPG: Deep Deterministic Policy Gradient
DL: Deep Learning
DLHRR: Deep Learning High‐Resolution Reconstruction
DTCO: Design–Technology Co‐Optimization
EDA: Electronic Design Automation
FL: Federated Learning
GAA: Gate All Around
GaAs: Gallium Arsenide
GaN: Gallium Nitride
GAN: Generative Adversarial Network
Ga_x_Oᵧ: Gallium Oxide with variable stoichiometry
GPT‐4: Generative Pre‐trained Transformer 4
GRU: Gated Recurrent Unit
ICDDM: Implicit Cross Domain Diffusion Model
IP: Intellectual Property
KDCDDM: Knowledge Distillation Cross Domain Diffusion Model
LED: Light‐Emitting Diode
LLM: Large Language Model
LSTM: Long Short‐Term Memory
MAP: Materials Acceleration Platform
ML: Machine Learning
NLVD: Nonlinear Variation Decomposition
OCD: Optical Critical Dimension
QML: Quantum Machine Learning

## 1 Introduction

For more than half a century, progress in semiconductor devices has been guided by geometric scaling laws, most notably Moore's law [1, 2, 3, 4, 5] and Dennard scaling [6, 7], which linked reductions in feature size to increases in integration density, performance, and energy efficiency [7]. Beyond lithography, these laws embodied an implicit engineering premise: physical iteration was inexpensive and readily available. New device concepts, materials, and processes could be, therefore, explored through repeated wafer fabrication, extensive TCAD sweeps, and destructive characterization, allowing engineering knowledge to accumulate primarily through physical trial and error. As long as physical iteration scaled with engineering ambition, increasing complexity translated naturally into technological progress.

This premise has progressively broken down at advanced technology nodes. In the sub‐3‐nm era, device architectures such as gate‐all‐around (GAA) nanosheets [8], heterogeneous material stacks [9], and 3D integration [10] have dramatically expanded the dimensionality of design and process spaces, while simultaneously driving up the cost, latency, and environmental footprint of physical iteration [11, 12]. Wafer‐based experimentation, high‐dimensional simulation sweeps, advanced metrology, and long qualification cycles now dominate development timelines and budgets. Under these conditions, simply increasing physical effort may no longer yield proportional gains, and classical geometric scaling laws start to cease to describe how progress is actually achieved in practice [13].

At the same time, artificial intelligence (AI) has begun to play a concrete role in alleviating the growing mismatch between escalating device complexity and the diminishing feasibility of physical iteration. Across semiconductor research, AI is reshaping how electronic materials, devices, and manufacturing systems are conceived and optimized [14, 15, 16, 17], particularly as conventional modeling and optimization struggle with high‐dimensional design spaces and complex variability at advanced nodes [18]. Data‐driven and physics‐informed models accelerate compact model development [19], parameter extraction [20], and inverse design in device simulation [21, 22], while reinforcement learning and digital twins support adaptive process control and predictive diagnostics in manufacturing [23, 24]. AI‐assisted virtual metrology enables critical parameters to be inferred from indirect measurements with reduced experimental overhead [25], and image‐based classification and anomaly detection enhance defect inspection, yield analysis, and reliability assessment [26, 27]. Beyond workflow acceleration, AI is also influencing materials discovery [28], device architecture exploration [29], and emerging computing paradigms such as neuromorphic and in‐memory systems [30], while extending into metrology and characterization through real‐time interpretation of spectroscopy and microscopy data. These developments indicate that AI is no longer merely improving efficiency locally but is progressively shifting semiconductor engineering away from exhaustive physical experimentation toward model‐driven inference—setting the stage for a more fundamental reorganization of how scaling can be sustained when physical iteration itself becomes the dominant bottleneck.

In this emerging regime, semiconductor progress can no longer be understood—or sustained—through the organizing principles of geometric scaling alone. We introduce **device‐level virtualization as a new scaling law**, not in the sense of further shrinking physical dimensions, but as a shift in the dimension along which progress scales, in order to support the development of academia and industry. Specifically, virtualization‐driven scaling reflects the expanding scope of engineering decisions that can be credibly supported by AI‐assisted virtual evidence rather than by exhaustive physical iteration. At early stages, virtualization enables a large space of the device design and modeling to be explored and pruned without proportional increases in simulation or fabrication effort. As it extends into fabrication and packaging, virtualization reshapes experimentation itself by narrowing process pathways and reducing reliance on split lots and trial‐and‐error. Ultimately, at the level of qualification, yield, and reliability, virtualization transforms how risk and readiness are assessed, allowing release decisions to be guided by layered, inference‐driven evidence rather than long physical validation cycles. Viewed collectively, semiconductor progress increasingly scales with how broadly virtual evidence can substitute physical iteration across the device lifecycle, decoupling innovation from the linear growth of cost, time, and resource consumption that has begun to constrain traditional scaling.

**FIGURE 1:**
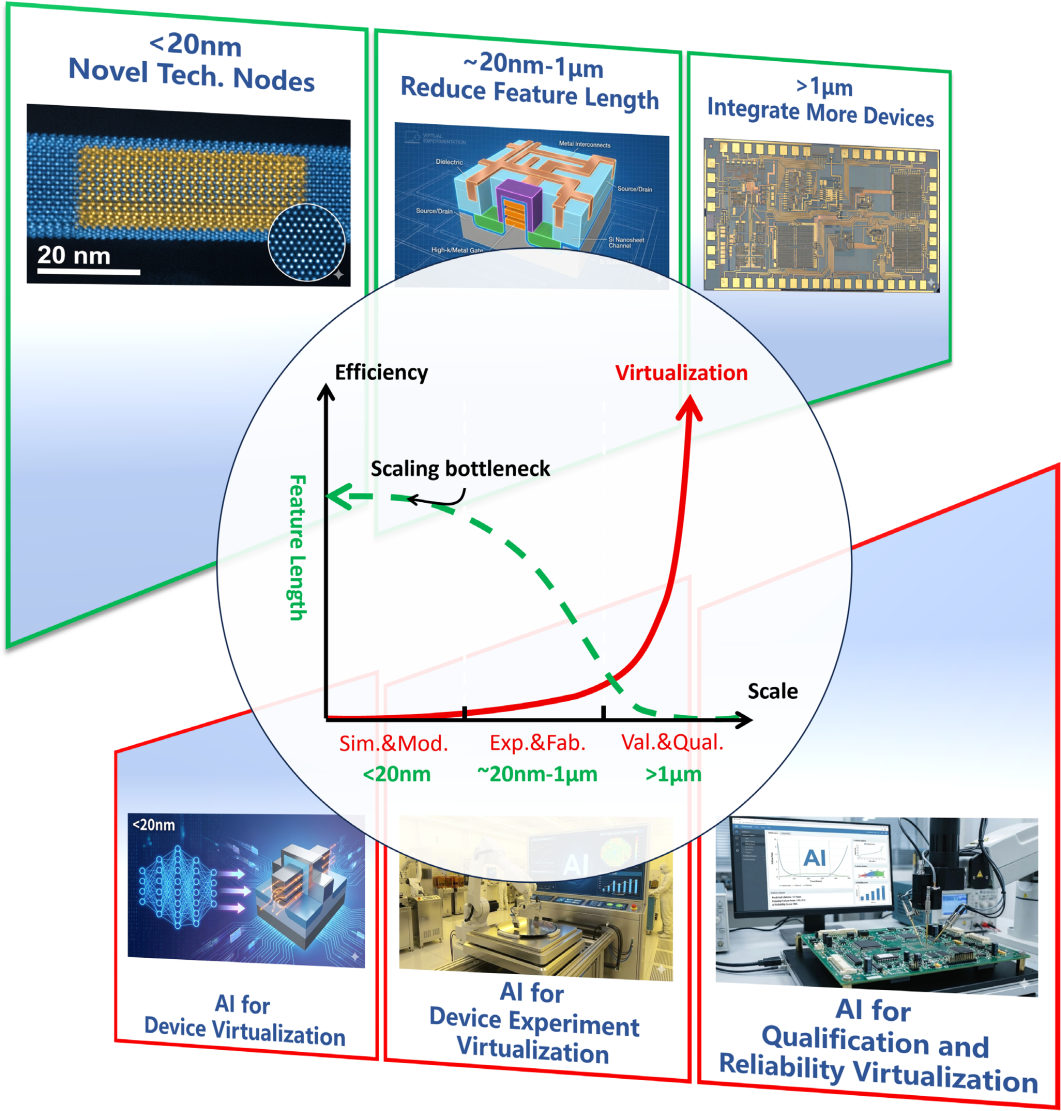
AI opportunities across the semiconductor device lifecycle and the shift toward virtualization‐driven scaling. The top row depicts the conventional direction of geometric scaling (right to left), where physical feature lengths shrink from >1 µm regimes (often emphasizing system‐level integration and “more devices”) to ∼20 nm–1 µm feature‐length reduction and further to <20 nm novel technology nodes (often focusing on new materials–device architectures). The central map highlights a growing scaling bottleneck: as geometric scaling and traditional efficiency gains increasingly collide with the rising cost, latency, variability, and environmental footprint of physical iteration, progress becomes less governed by geometry alone (green line). In parallel, AI‐enabled virtualization and virtual evidence (red line) increase in importance across the development scale—linking simulation and modeling, experimentation and fabrication, and validation and qualification—and enabling engineering decisions to be supported by inference rather than exhaustive trial‐and‐error. The bottom row summarizes three corresponding levers of this transition: AI for device virtualization (surrogate and physics‐informed modeling, parameter extraction, inverse design, etc.), AI for experiment virtualization (digital twins, reinforcement‐learning–assisted process optimization, virtual metrology, etc.), and AI for qualification and reliability virtualization (defect inference, variability/yield modeling, predictive diagnostics, etc.), together, as an up‐scaling trend, illustrating how AI can reorganize semiconductor development around credible virtual evidence when physical iteration becomes the dominant constraint. Gemini generated art components of this figure.

In this Perspective, as shown in Figure 1, we argue that the most consequential role of AI in semiconductor devices is not incremental acceleration, but the virtualization of engineering progress itself. Rather than reviewing AI methods or application domains in isolation, we focus on how virtualization reshapes the logic by which engineering progress of semiconductor devices is generated, evaluated, and scaled under increasing physical constraints. We examine how **virtualized experimentation, measurement, qualification, and decision‐making** collectively alter the balance between physical experiments and model‐based inference across the device lifecycle, and how this shift redefines the role of AI from a performance enhancer to a structural enabler of sustainable progress. Particular attention is paid to the conditions under which virtual evidence can credibly substitute physical iteration, including the roles of, e.g., fabrication short‐loops, quantification, interpretability, and validation. By framing diverse AI‐enabled advances within a virtualization‐driven scaling regime, this Perspective aims to clarify not only where AI is most impactful, but also where its limits lie, providing a coherent lens for understanding how semiconductor device innovation can continue beyond the exhaustion of traditional geometric scaling.

## 2 AI to Enable Device Virtualization

### 2.1 Virtualizing Device Design and Modeling

**FIGURE 2:**
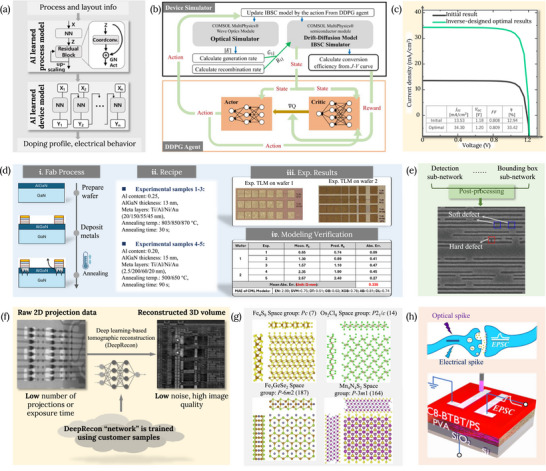
Some recent advancements of AI for semiconductor devices that enable the scaling of virtualization. a) schematic diagram of an AI‐based emulator (Real‐Time Twin, RTT) designed to emulate the TCAD process and device simulation. Adapted with permission [34]. b) Flow chart of an inverse design simulator with a drift‐diffusion device simulator, and c) the current‐voltage curve for the device structure with initial device parameter and the inverse‐designed optimal device parameters by using a joint drift‐diffusion simulator and deep RL scheme. Adapted with permission [21]. d) i and ii show the experimental setup, metal stacks were processed to form Ohmic contacts under various annealing conditions. iii exhibits the optical microscopy image of the fabricated devices. iv summarizes the measured and predicted contact resistance values for each sample, along with the corresponding absolute errors. Adapted with permission [15]. e) shows the artificially generated layout and SEM images. Adapted with permission [27]. f) schematics of the architecture of the deep learning high‐resolution reconstruction (DLHRR) method, which is used to generate high‐quality images. Adapted with permission [49]. g) shows the top and side views of the structure of 2D ferromagnetic materials generated by ML regression, i, Fe_4_S_8_, ii, Os_2_Cl_8_, iii, Fe_3_GeSe_2_, iv, Mn_4_N_3_S_2_. Adapted with permission [17]. h) Schematic of a biological synapse and the geometry of the three‐terminal artificial synapse. Adapted with permission [50].

Device exploration has traditionally relied on extensive fabrication‐based experimentation and exhaustive TCAD sweeps to navigate the strong sensitivity of device performance to geometry, contacts, doping, material composition, etc. As device feature length shrinks to the quantum regime and device architecture grows more complex, this paradigm becomes increasingly untenable due to the combinatorial expansion of design spaces and the rising cost of physical and numerical iteration. In response, as shown in Figure 2, device design should progressively be virtualized through AI‐assisted frameworks that enable large regions of the design space to be screened, constrained, or excluded without direct physical realization, while still respecting underlying device physics [15, 31, 32].

A preliminary manifestation of this shift is the emergence of AI‐enabled inverse [22, 33] and surrogate‐driven exploration [34] strategies, which allow design hypotheses to be tested virtually before committing to fabrication or full‐scale simulation. In photonic devices, for example, where the device response can be parameterized with relatively well‐defined physical models, inverse design approaches have optimized architectures and exceeded target efficiency benchmarks with substantially fewer physical experiments, illustrating how virtual experimentation can replace trial‐and‐error exploration [35, 36]. In addition, Bayesian optimization and related probabilistic frameworks play a key role in this process by guiding exploration across high‐dimensional parameter spaces, such as bandgap grading or layer thicknesses, with quantified uncertainty rather than exhaustive sampling [37, 38].

Virtualization of device design has been further hinted by advances in AI‐assisted modeling and simulation that reduce dependence on computationally intensive TCAD workflows. Physics‐informed neural networks (PINNs) embed drift–diffusion equations and physical constraints directly into learning architectures, enabling device behaviors to be inferred without repeatedly solving full numerical models [39]. Hybrid frameworks that couple physics‐based TCAD with machine learning act as virtual experiment engines, emulating device responses in real time and enabling large‐scale parametric studies that would otherwise be impractical [34]. Neural‐network‐based and graph‐based compact models similarly virtualize device behavior by capturing process variation and layout dependencies with far fewer simulations, thereby shortening design cycles while preserving essential physical trends [40]. Reinforcement learning and Bayesian optimization further close the loop by enabling device structures to be optimized through virtual interaction with surrogate models, achieving performance gains with substantially reduced experimental and simulation effort [21, 23].

Virtualization also holds great potential in device–material co‐exploration, where material synthesis and device performance are linked through model‐mediated inference rather than sequential experimentation. E.g., surrogate learning frameworks have been employed to design electronic materials such as GaO films with tailored bandgaps for power electronics, while active learning loops guide magnetron sputtering conditions to achieve target properties without exhaustive trial fabrication [16]. In parallel, AI‐based surrogates can also enable rapid prediction of terahertz device characteristics, supporting high‐frequency device development with orders‐of‐magnitude reductions in computational cost [41].

These developments suggest a shift in which progress depends less on the scale of physical or numerical iteration and more on the ability to virtualize exploration. As surrogate, physics‐informed, and inverse‐design frameworks mature, large regions of design space can be prioritized or excluded before committing to fabrication or exhaustive TCAD sweeps [42]. Large language models can further structure and reuse published experimental knowledge as virtual evidence, reducing redundant experiments [14]. As a result, physical runs increasingly serve targeted validation and calibration rather than broad exploration.

### 2.2 Virtualizing Device Experimentation

If design and modeling become virtual‐first (Section 2.1), the next transition is virtualizing experimentation in fabrication and packaging, where progress has relied on split lots and repeated tuning. At advanced nodes, iteration is limited less by what can be designed than by what can be executed within cost, schedule, and sustainability constraints. Virtualized experimentation shifts iteration from wafer‐based trial‐and‐error to model‐mediated exploration that narrows, stress‐tests, and optimizes candidate process paths before physical commitment. Importantly, this does not “replace manufacturing”; it replaces unstructured process learning with virtual evidence that prioritizes which experiments to run, in what order, and with what expected risk. Digital twins, surrogates [51], and reinforcement learning [52, 53] support this shift by proposing policies and evaluating trade‐offs, while physical runs increasingly act as validation checkpoints [54, 55].

Hence, virtualizing fabrication and packaging experimentation should center on one question: how to obtain process knowledge without paying for it entirely in wafers, facilities, and iterations. This requires a “virtual eye” that can infer process states and outcomes from indirect signals, simulations, and historical data, allowing learning to proceed without executing every physical trial. In complex fabrication sequences, there already exist virtual metrology‐style predictors built on indirect sensing streams or computer vision (e.g., all kinds of AI‐based spectroscopy). The “virtual eye” can forecast process outcomes quickly enough to support in‐line decisions and reduce the need for repeated split‐lot trials—functioning as virtual experiment proxies that guide which knobs matter and where to intervene [56]. Complementarily, reinforcement learning supports the virtualization of experimentation by detecting and learning control‐behavior strategies through simulated interaction with a process environment; for instance, RL‐based feedback control, e.g., in optical proximity correction (OPC), has been reported to reduce iterations and runtime substantially, effectively replacing repeated manual tuning cycles with policy search in a virtual loop [57, 58].

At a higher level, virtualization extends beyond isolated tools into an integrated experimental organism, in which AI‐driven digital twins function as a “virtual body” that emulates fabrication and packaging processes, while virtual assistants and optimization engines act as a virtual brain that interprets outcomes, proposes actions, and coordinates decision‐making. Together, they enable end‐to‐end virtual experimentation by integrating heterogeneous manufacturing information to streamline process debugging and accelerate design–technology co‐optimization (DTCO) [59, 60]. When process variables can be represented in structured forms, multi‐objective Bayesian optimization provides a concrete mechanism for this virtual brain to explore fabrication chemistry and integration trade‐offs—for example, guiding wet etching bath design and device‐structure adjustments to achieve uniform etching and improved stability with far fewer physical trials [61]. Across these approaches, in other words, the unifying shift is that “experimenting” increasingly refers to searching, screening, and packaging candidate process paths within a coupled “virtual body–brain” system, while wafers are reserved primarily for validation rather than exploration.

Notably, recent advances in advanced packaging already reflect key elements of the proposed virtual eye–body–brain paradigm, even if they are not explicitly articulated in this form [62]. In complex packaging processes dominated by thermo‐mechanical coupling and heterogeneous material interactions, data‐driven warpage prediction frameworks effectively provide a virtual eye, enabling latent deformation states to be inferred from limited manufacturing and simulation data rather than exhaustive physical measurement. Hybrid modeling approaches that integrate finite‐element simulations with machine learning act as a virtual body, emulating nonlinear packaging responses under constrained data conditions. Optimization and parameter‐feedback mechanisms built on these predictive models further serve as a virtual brain, supporting informed design and process decisions without repeated physical builds. The demonstrated effectiveness of such AI‐assisted warpage prediction and feedback mechanisms in advanced packaging—one of the most nonlinear and data‐constrained domains in semiconductor manufacturing—provides early empirical support for virtualization as a viable experimental paradigm rather than a purely conceptual proposal.

**FIGURE 3:**
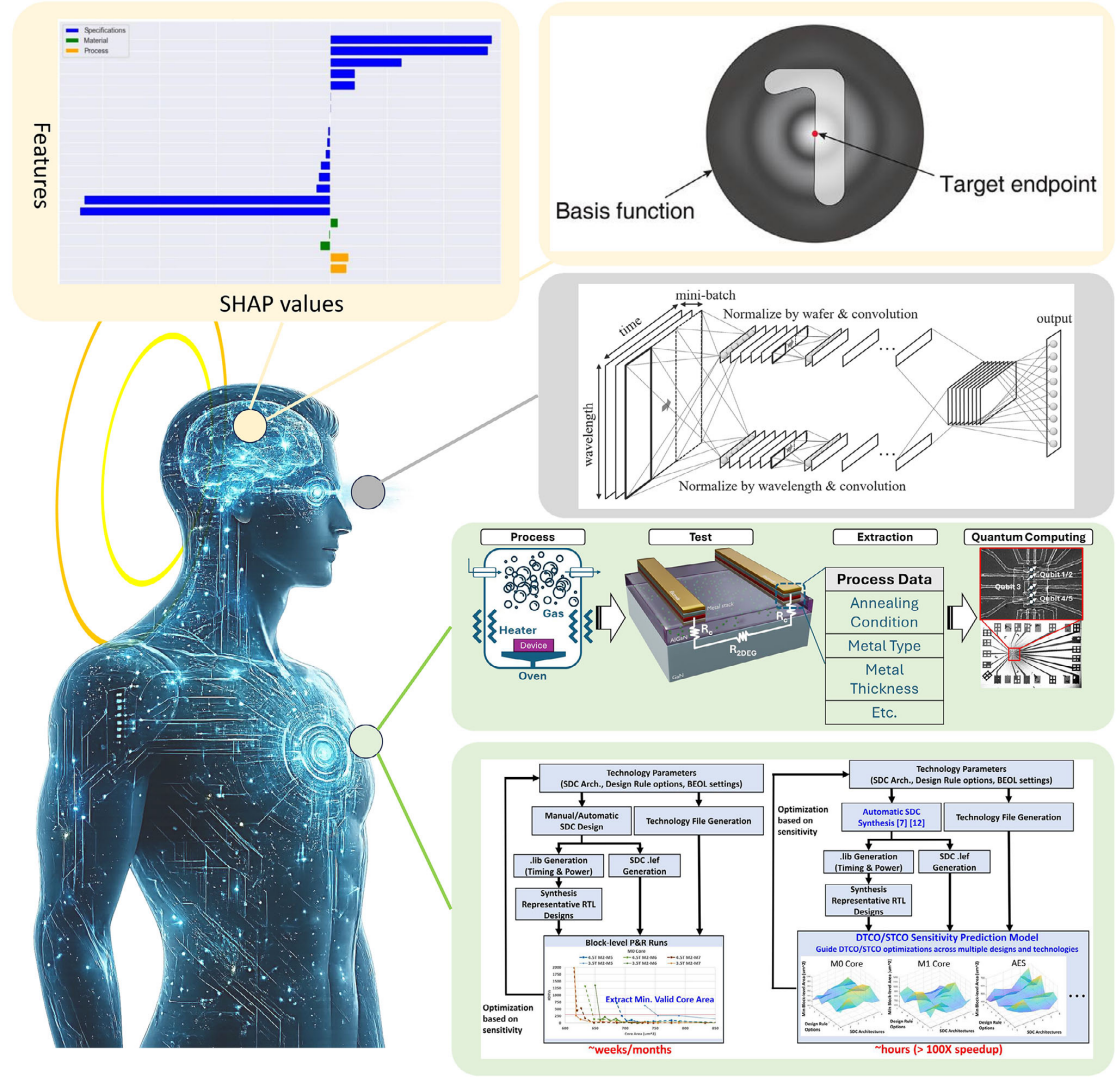
AI‐enabled virtualization for Device Experimentation including fabrication and packaging: virtual eyes, virtual body, and virtual brain. Virtual eyes emulate metrology and inspection through data‐driven perception: deep learning on optical emission spectroscopy enables virtual metrology. Virtual body captures process–device–package co‐optimization via surrogate and optimization engines. Virtual brain represents the decision layer that closes the loop from prediction to actionable design choices. Together, these examples illustrate how AI progressively shifts iteration from costly physical loops toward scalable, inference‐driven virtualization across manufacturing and packaging workflows. Inset adapted with permission [15, 56, 57, 60, 62]. Gemini generated art components of this figure.

Collectively, as shown in Figure 3, these advances point toward a qualitatively new form of device experimentation, in which virtualization emerges as an integrated virtual human‐like intelligence (virtual eye–body–brain system) rather than a collection of isolated tools. In this regime, the virtual eye infers process states from indirect signals, the virtual body emulates fabrication and packaging responses through digital twins, and the virtual brain actively explores, evaluates, and refines candidate process paths via learned control and optimization policies. Fabrication and packaging thus evolve toward a virtual‐first experimentation stack, where learning throughput is governed less by the number of split lots and more by the fidelity, adaptivity, and trustworthiness of virtual evidence that can safely narrow the process window before any wafer is processed. Looking forward, it has been suggested that scalable manufacturing workflows will increasingly rely on interactive, and potentially embodied, virtual experimentation—systems capable of autonomously proposing, executing, and revising experiments in virtual space—while physical runs are reduced to sparse, information‐rich probes for calibration and validation. More radically, drawing inspiration from parallel developments in other research domains—such as biotechnology, where virtual cell models are increasingly used to explore and test hypotheses [63]—in this paradigm experimentation itself becomes a cloud‐executed, continuously learning process. Manufacturing knowledge acquisition is thereby fundamentally decoupled from the linear cost of wafers, facilities, and iteration cycles.

### 2.3 Virtualizing Qualification and Reliability

If device design (Section 2.1) and experimentation (Section 2.2) can be progressively virtualized, the final—and most consequential—stage is the virtualization of qualification, yield, and reliability. These stages ultimately determine whether a device or technology is manufacturable at scale, and therefore represent the widest scope of engineering decision‐making across the device lifecycle. Traditionally, such decisions have relied on long qualification cycles, exhaustive characterization, and conservative guard‐banding, with reliability established through accumulated physical evidence under stress and variation. As materials systems, device architectures, and packaging technologies continue to grow in complexity, however, this physically exhaustive approach becomes increasingly misaligned with development timelines, cost constraints, and sustainability requirements.

In response, AI is transforming qualification from a process dominated by delayed physical confirmation into one guided by virtual evidence—model‐mediated signals that enable early screening of yield and reliability risks. A primary manifestation of this shift is the virtualization of defect‐ and fault‐related evidence. Deep learning models, including adversarial architectures, achieve high accuracy in detecting and classifying hard and soft defects from industrial inspection data [27, 64], allowing defect images to be translated into standardized inputs for yield‐risk assessment rather than treated as isolated observations. Weakly supervised approaches based on diffusion models further reduce annotation requirements by enabling defect localization and segmentation without dense labels, improving scalability across products and process nodes [67, 68, 69]. Federated learning combined with explainable AI (XAI) extends these capabilities to distributed manufacturing environments, enabling decentralized fault detection while preserving data privacy and providing interpretable diagnostic rationale [70]. Collectively, these approaches shift qualification logic from retrospective inspection toward proactive, inference‐driven screening.

The scope of virtualization further expands beyond wafer‐level fabrication into system‐level reliability contexts. Machine‐learning‐based fault diagnosis applied to multilevel inverters and power devices operating under complex electrical conditions enables likely failure modes to be inferred from operational signatures rather than from catastrophic outcomes [71]. Predictive failure analysis leverages machine learning and image processing to anticipate defects upstream, allowing corrective actions before reliability degradation propagates through the manufacturing chain [72]. In parallel, data‐driven models that correlate tool‐ and chamber‐level signatures with yield outcomes enable device‐level yield optimization with reduced experimental overhead in other device types, providing an additional layer of virtual evidence to support qualification and release decisions [26, 56, 73]. At this level, virtualization no longer targets individual defects or parameters, but supports integrative judgments about manufacturability and risk.

At the characterization level, virtualization manifests as a transition from direct, exhaustive measurement toward inference‐rich evidence generation. Deep learning–based segmentation and super‐resolution methods enhance the efficiency and consistency of electron microscopy and white‐light interferometry analysis [74, 75], while AI‐powered reconstruction accelerates 3D X‐ray tomography for advanced package failure analysis with fewer measurements and reduced manual effort [76]. Similar trends are observed in spectroscopy and scattering techniques: data‐driven or machine‐learning‐enhanced total reflection X‐ray fluorescence (TXRF) is expected to improve detection sensitivity and shorten measurement times for contamination control [77, 78, 79], and recent progress in AI‐assisted optical critical dimension (OCD) modeling can also be utilized to extract structural parameters from increasingly complex device geometries [80]. In all these cases, characterization outputs increasingly function as virtual proxies for qualification‐relevant information rather than as stand‐alone measurements.

On a batch‐production scale, AI‐driven virtualization fosters a synergy between operational efficiency and sustainable manufacturing. AI‐enhanced simulation and virtual screening have been shown to dramatically reduce material consumption, chemical usage, and associated carbon footprints by minimizing unnecessary experiments [16]. AI‐augmented life cycle assessment further improves transparency and decision‐making across semiconductor supply chains, enabling qualification strategies that account for environmental impact alongside technical risk [45, 80, 81]. Predictive maintenance and AI‐driven fault detection reduce waste and energy loss by preventing defective batches and unplanned downtime [70, 72, 82], while autonomous material and device acceleration platforms (AMADAP) embed AI‐guided, closed‐loop learning into qualification workflows to accelerate convergence with fewer resource‐intensive iterations [83, 84] Tables 1, 2, 3.

**TABLE 1:** AI‐enabled virtualization attempts for device design, simulation, and modeling.

Virtualization	AI method	Contribution	Refs.
Virtual designer	GPT‐4 and Extreme Gradient Boosting	Developed an AI agent integrating LLM and ML for efficient extraction and modeling of OFET device parameters to guide device design optimization	[14]
	Deep Learning model optimized by Genetic Algorithm	Automated DL model design for semiconductor device parameter prediction	[43]
Virtual TCAD engineer	Automated data generation framework with TCAD and ML	Framework assures reliable ML models for semiconductor process and device optimization	[20]
	Computer vision and image processing algorithms	Developed computer vision‐based metric for validating semiconductor device fabrication simulations	[24]
	Hybrid AI and physics‐based modeling	Developed methodologies bridging TCAD and AI for efficient semiconductor device/process modeling	[34]
	Multi‐fidelity modeling and active learning	Developed efficient NN‐based compact model reducing SPICE simulation time for advanced device modeling	[40]
	Machine Learning with Explainable AI (Shapley values)	Proposed ML framework that learns process variations efficiently and provides interpretable insights surpassing TCAD simulators	[44]
	Quantitative analysis of simulation and AI impact	Quantified environmental benefits of computational simulation and AI in semiconductor device/process optimization	[45]
	Graph Attention Network (RelGAT) with graph‐based universal encoding	Proposed AI‐driven EDA framework for device‐level modeling integrating graph representations and TCAD data	[46]
	3D finite‐element quantum‐corrected semi‐classical/classical simulation and ML‐based prediction	Provided comprehensive software suite combining physics‐based simulation and ML for advanced device modeling	[47]
	3D CNN	3D simulation accelerated by 3D CNN	[48]

**TABLE 2:** AI‐enabled virtualization attempts for fabrication and packaging.

Virtualization	AI method	Contribution	Refs.
Virtual eyes	DL with optical emission spectroscopy	Realizing virtual metrology in semiconductor manufacturing	[56]
	ML‐guided optical proximity correction	Fast, accurate, and manufacturable curve detection and correction	[57]
	Deep RL	Photolithographic mask optimization	[58]
	ML with image enhancement and Explainable AI	Applied ML and XAI for enhanced defect detection and process optimization in semiconductor manufacturing	[64]
	1D Convolutional Autoencoder with Localized Reconstruction Error	Proposed interpretable deep learning approach for semiconductor process anomaly detection	[65]
	Graph attention network	Computer vision‐assisted DTCO	[46]
Virtual body	Bootstrap aggregation and gradient boosting	ML‐enhanced DTCO	[60]
	Multi‐objective Bayesian optimization	Optimal design of wet etching bath for 3D flash memories	[61]
	Explainable AI (XAI) and Virtual Metrology with status‐variable identification	Developed high‐performance VM models with XAI for precise semiconductor plasma process control	[66]
Virtual brain	Genetic algorithms and ANNs	High‐accuracy semiconductor package warpage estimation	[62]

**TABLE 3:** AI for device qualification and reliability.

AI method	Features to be used in virtualization	Refs.
GPT‐4 and Extreme Gradient Boosting	Integrated LLM with ML for automated extraction and device design guidance in organic optoelectronics metrology and characterization	[14]
Deep learning with scientific knowledge integration	Enhanced process and device modeling for semiconductor metrology and characterization	[18]
CNN‐based resist model training with adaptive learning rate and gradient clipping	Advanced resist modeling for improved lithography metrology	[19]
Automated data generation framework for ML training	Framework enables actionable ML models for semiconductor device process control	[20]
Reinforcement learning (PPO and A2C) agents	Accelerated design exploration for power semiconductor device metrology	[23]
Computer vision with image processing and pattern matching	Tool for validating semiconductor device fabrication and simulation accuracy	[24]
Weighted Transfer Lasso regression	Enabled fast VM model adaptation for new semiconductor manufacturing equipment	[25]
Deep learning with conditional GAN and Focal Loss	Improved yield and productivity via accurate and efficient defect detection	[27]
Federated Learning (FL) and Explainable AI (XAI)	Developed privacy‐preserving, transparent fault detection model for distributed semiconductor manufacturing data	[70]
Virtual Metrology (VM) with AI and Explainable AI (XAI)	High‐performance VM models for precise process control in semiconductor plasma processes	[66]
Machine learning with image enhancement and Explainable AI	Enhanced defect detection and process control in semiconductor lithography	[64]
AI‐based image segmentation using minimal annotated training images	Enabled automated characterization workflows for semiconductor device structure inspection	[67]
Neural network classification	Provided insights into the physical parameters underlying GaAs defect products	[85]
Multi‐layer perceptron	ML‐guided curvilinear OPC	[57]
Weakly supervised diffusion models (ICDDM, CDLDM, KDCDDM) with GAN	Provided efficient and effective defect detection solution for semiconductor production	[86]
Nonlinear Variation Decomposition (NLVD) of Neural Networks	Provided quantitative evaluation of process defects and mechanisms per sample and wafer for enhanced causal analysis	[87]
Deep learning with weak supervision and active learning	Reduced cost and accelerated analysis of TEM images for semiconductor device metrology	[68]
Generative AI model	Enabled proactive CMP defect detection and process optimization in semiconductor manufacturing	[88]
Multilayer perceptron with pre‐trained variational auto‐encoder initialization	Enabled virtual diagnostics for plasma monitoring in semiconductor manufacturing	[73]
YOLOv5 with transformer module (LyFormer model)	Enhanced accuracy and speed for semiconductor part defect detection in production lines	[72]
YOLOv5 with transformer and label normalization	Applied AI for precise component quantification in semiconductor manufacturing execution systems	[89]
Material‐device‐algorithm co‐design with organic field‐effect transistor array and reservoir computing	Developed AI‐enabled hardware for selective and accurate gas metrology	[90]
Machine vision and AI recognition	Provided AI‐based damage identification for LED reliability metrology	[82]
Supervised machine learning with LSTM and GRU	Provided accurate ML‐based GaN device models for power electronics characterization	[91]

These developments indicate that qualification, yield, and reliability are transitioning from physically exhaustive validation toward virtual evidence–driven decision‐making, representing the broadest and most integrative level of virtualization‐driven scaling. It can be predicted with increasing confidence that future qualification frameworks will rely on layered virtual signals—defect inference, variability modeling, and predictive reliability assessment—to screen risk and guide release decisions, while physical stress testing is progressively reserved for calibration, compliance, and rare failure modes. In this emerging regime, reliability itself becomes a virtualizable engineering quantity, allowing semiconductor progress to scale with the scope and fidelity of qualification models rather than with the duration and cost of physical qualification cycles.

## 3 Challenges and Roadmap for Virtualization‐Driven Scaling

Despite rapid progress, virtualization‐driven scaling is neither automatic nor unconstrained. Its effectiveness is ultimately limited by the conditions under which virtual evidence can credibly substitute physical iteration. Several fundamental challenges therefore define the current and future boundaries of virtualization across the semiconductor device lifecycle.

1. **Trust and epistemic uncertainty**: The core problem is not point accuracy but trust [92]. As virtualization moves from local optimization to qualification and release decisions, errors can propagate across layers. Models often perform well in interpolation but fail under extrapolation, rare events, or distribution shifts. Uncertainty quantification, calibration, and interpretability are therefore required [93]—especially where decisions have irreversible economic consequences.
2. **Coherence across stages**: Current efforts are fragmented: design models, process surrogates, defect classifiers, and reliability predictors are rarely coupled [92, 93]. Without consistent propagation of information and uncertainty, virtualization remains a set of local accelerators rather than a scaling principle.
3. **Sustainability and feedback bottlenecks**: Virtualization reduces wafer iteration but introduces non‐trivial compute cost [94, 95]. Meanwhile, sparse or delayed physical validation slows model updates and drift detection, creating a structural tension between fewer wafers and continual grounding.
4. **Security, integrity, and governance**: As virtual evidence becomes central to qualification and release, provenance, auditability, and secure handling become first‐order requirements [96]. Data leakage, poisoning, untracked updates, or lost provenance can directly undermine engineering decisions, not just model performance.

To function as a sustainable scaling law rather than a collection of isolated accelerators, virtualization must expand in a controlled and hierarchical manner, with each stage explicitly addressing the constraints identified above. The roadmap for virtualization‐driven scaling is therefore defined not by chronological milestones, but by the progressive enlargement of the engineering decision scope that can be credibly supported by virtual evidence.

### 3.1 Stage I: Local Virtualization with Bounded Confidence

Near‐term progress focuses on strengthening virtualization at local decision levels, such as device design exploration, parameter screening, and surrogate‐based modeling. At this stage, virtualization is primarily interpolative, operating within well‐characterized regimes where physics‐based priors, data availability, and validation feedback are relatively strong. The central objective is not maximum accuracy, but bounded trust: rigorous uncertainty quantification, calibration, and interpretability must accompany virtual predictions to prevent overconfidence. Physics‐informed learning, multi‐fidelity modeling, and uncertainty‐aware surrogates serve as foundational enablers, allowing virtual evidence to accelerate exploration without yet dominating decision‐making.

### 3.2 Stage II: Coherent Virtualization Across Design and Experimentation

The next scaling step requires overcoming fragmentation across lifecycle stages. Virtualization must transition from isolated models toward coherent, cross‐stage decision frameworks that couple device design, fabrication, and packaging experimentation. Here, virtual eye–body–brain systems begin to emerge, integrating perception (inference from indirect signals), emulation (digital twins and hybrid models), and decision‐making (optimization and control). Physical experiments are no longer routine drivers of learning, but become sparse, information‐rich anchors that efficiently update and correct virtual models. Scaling at this stage depends critically on reducing the cost of updating the model and ensuring consistent propagation of trusted data across components.

### 3.3 Stage III: Virtualization of Qualification and Risk Assessment

Once coherence is established across design and experimentation, virtualization can expand into qualification, yield, and reliability decisions. At this stage, the virtualizable object is no longer a parameter or process response, but engineering itself. Separated virtual components—defect inference, variability modeling, stress‐response prediction, and degradation forecasting—are combined to support integrative engineering judgments about manufacturability and release readiness. Physical stress testing and long qualification cycles are progressively reserved only for calibration, compliance, and rare failure modes. Scaling is achieved by shifting the dominant evidence base for release decisions from accumulated separated physical trials to inference‐driven virtual engineering evidence with explicit confidence bounds.

### 3.4 Stage IV: Governed and Sustainable Virtualization Ecosystems

As virtualization reaches qualification‐level decision scope, security, intellectual property protection, and governance become first‐order scaling constraints. Virtual evidence must be traceable, auditable, and securely managed across distributed teams and infrastructures, ensuring integrity and provenance throughout the device lifecycle. In parallel, sustainability considerations impose constraints on computational cost, model complexity, and update frequency. At this stage, virtualization‐driven scaling succeeds only if computational efficiency, energy consumption, and environmental impact are co‐optimized alongside engineering objectives. Cloud‐executed experimentation, shared model infrastructures, and standardized governance frameworks enable virtualization to scale without introducing new systemic risks.

### 3.5 Long‐Term Steps: Virtualization as an Organizing Principle

Ultimately, virtualization‐driven scaling converges toward an engineering regime in which experimentation, qualification, and decision‐making are predominantly executed in virtual space, while physical fabrication provides periodic grounding rather than continuous iteration. Progress is no longer limited by wafer availability or qualification cycle length, but by the fidelity, integration, and governance of virtual evidence. In this regime, scaling is sustained not by shrinking geometry, but by expanding the scope of engineering decisions that can be made without proportional physical cost.

**FIGURE 4:**
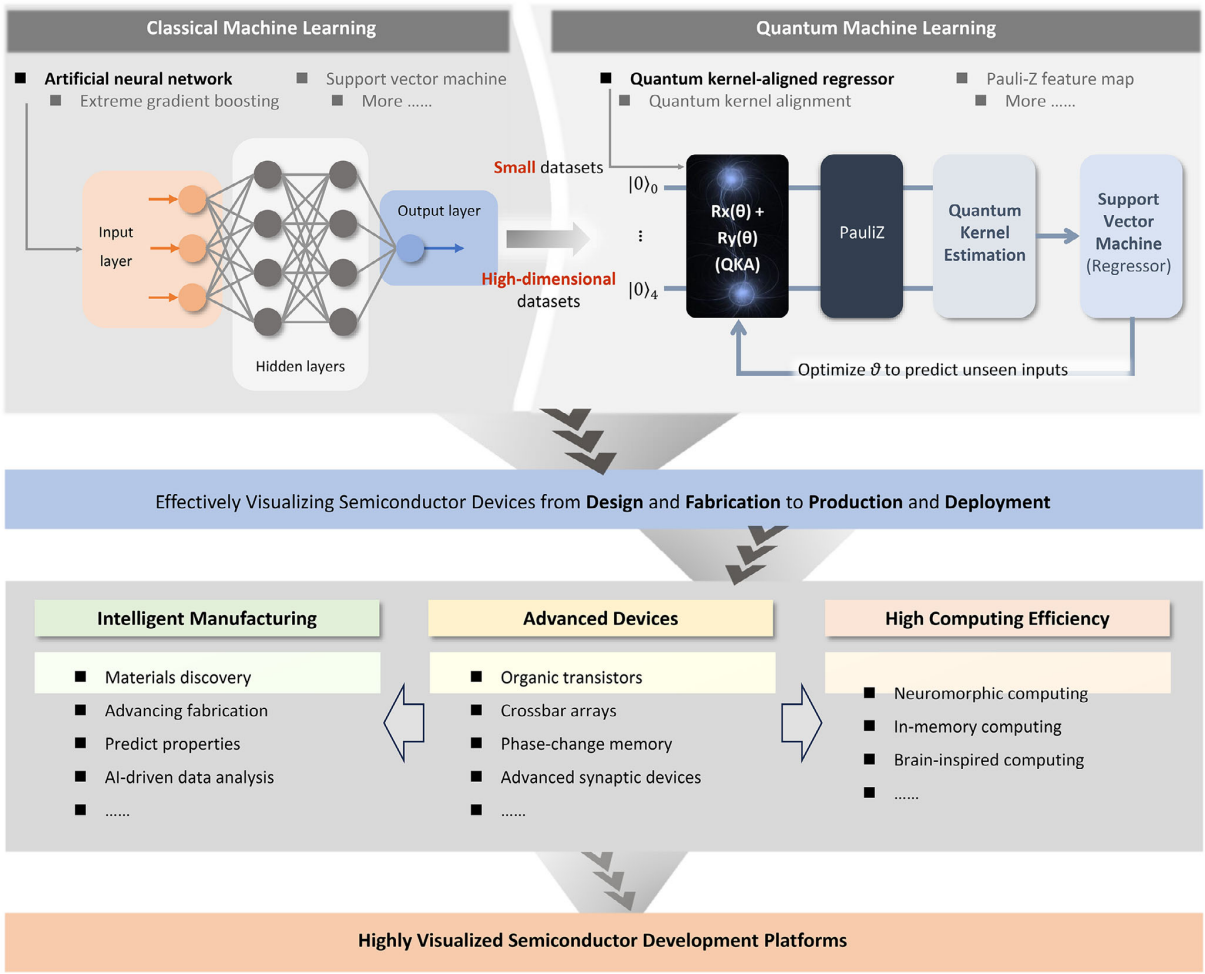
Roadmap implementation example: QML‐enabled virtualization as a building block of virtualization‐driven scaling. Here we provide a schematic example of how quantum machine learning (QML)—here illustrated with a quantum‐kernel regressor [15, 97]—could be integrated into a virtualization workflow as one candidate mechanism for generating virtual evidence under small‐data constraints. Using a quantum feature map and kernel estimation (e.g., via Pauli‐Z measurements), the workflow constructs a task‐aligned kernel that is then used by a classical regressor (e.g., support vector regression). The purpose of the example is illustrative rather than benchmark‐driven: it shows how a kernel‐based QML module can be embedded within a broader lifecycle pipeline that links design/modeling, fabrication/experimentation, and qualification/decision support. In this sense, the figure demonstrates how AI‐enabled virtualization can reorganize engineering iteration around inference and targeted validation, rather than exhaustive physical trial‐and‐error.

For practical implementation, Table 4 provides a concise checklist of common challenges and corresponding mitigation strategies for AI‐enabled virtualization, serving as a quick reference for readers. Figure 4 illustrates, at a schematic level, how quantum computing could be used to implement the roadmap in a small‐dataset scenario.

**TABLE 4:** Common challenges and practical mitigation strategies for AI‐enabled virtualization.

Challenge	Typical failure mode	Practical mitigation for Virtualization
Small‐sample regimes	Overfitting; unstable rankings	Physics‐informed priors; transfer learning; active learning; Quantum machine learning
High‐dimensional design/process spaces	Combinatorial explosion	Surrogate‐assisted optimization; dimensionality reduction with physical constraints or quantum technology; Bayesian optimization with safety margins
Domain shift (tool, node, material, fab)	“Works in one fab, fails in another”	Domain adaptation; hierarchical/multi‐task learning; causal feature selection; periodic re‐calibration with drift monitoring
Synthetic/TCAD data fidelity	Sim‐to‐real gap	Discrepancy modeling; hybrid training with small real anchor sets; sensitivity‐aware validation experiments
Interpretability/traceability	Black‐box recommendations	Explainable AI (feature attribution, counterfactuals); model cards; audit trails; constraint‐based optimization
Reliability of virtual evidence	Miscalibrated confidence	Calibrated uncertainty (ensembles/Bayesian surrogates); conformal prediction; staged validation ladders
Sustainability/compute footprint	Shifting burden to compute	Measure and report energy/CO_2_; co‐design models for efficiency; reuse pretrained models; avoid redundant retraining

## 4 Conclusion and Outlook

This Perspective has argued that the most consequential impact of artificial intelligence on semiconductor devices is not incremental acceleration of existing workflows, but the emergence of **virtualization as a new scaling law**. As geometric scaling approaches physical, economic, and sustainability limits, progress increasingly depends on how broadly engineering decisions can be supported by **virtual evidence** rather than exhaustive physical iteration. Across the device lifecycle—from design and modeling, through fabrication and packaging experimentation, to qualification and reliability—AI enables a systematic shift from trial‐and‐error experimentation toward inference‐driven exploration, screening, and risk assessment.

We have shown that virtualization is already taking concrete form at multiple levels. In device design and modeling, surrogate learning, and inverse design frameworks allow large regions of design space to be explored without proportional increases in simulation or fabrication effort. In experimentation, digital twins, virtual metrology, and reinforcement learning move the locus of iteration from wafers to virtual environments, transforming physical experiments into targeted validation checkpoints rather than primary drivers of learning. At the level of qualification and reliability, AI‐based defect inference, variability modeling, and predictive diagnostics increasingly provide early, integrative signals of manufacturability and risk, reshaping how release decisions are made under uncertainty.

Taken together, these developments suggest that semiconductor progress is no longer governed solely by how small devices can be made, but by how effectively **physical iteration can be virtualized, structured, and constrained**. Virtualization‐driven scaling decouples innovation from the linear growth of cost, time, and resource consumption that now constrains traditional scaling laws. In this sense, AI functions not merely as a performance enhancer, but as a structural enabler that reorganizes the logic of semiconductor engineering itself.

Looking forward, the central question is not whether virtualization will expand, but **under what conditions it can credibly function as a sustainable organizing principle for semiconductor progress**. The long‐term trajectory of virtualization‐driven scaling will be shaped by three tightly coupled dimensions: trust, coherence, and governance.

First, trust in virtual evidence must be explicitly engineered rather than implicitly assumed. As virtualization expands toward qualification‐ and release‐level decisions, uncertainty quantification, calibration, and interpretability become first‐order requirements. Future advances are likely to emphasize uncertainty‐aware surrogates, multi‐fidelity modeling, and physics‐informed learning that expose—not obscure—the limits of inference. Virtual evidence will increasingly be judged not by point accuracy alone, but by whether its confidence bounds are reliable enough to support irreversible engineering and economic decisions.

Second, virtualization must evolve from fragmented tools into **coherent, lifecycle‐spanning decision frameworks**. The greatest scaling gains will not arise from isolated improvements in modeling or inspection, but from the integration of virtual eyes, bodies, and brains into unified systems that propagate information and uncertainty consistently across design, fabrication, and qualification. Reducing feedback latency between virtual predictions and sparse physical validation will be critical, particularly as process complexity, heterogeneity, and time‐dependent degradation continue to grow.

Third, governance, security, and sustainability will define the ultimate ceiling of virtualization‐driven scaling. As virtual evidence becomes central to qualification and release decisions, its integrity, provenance, and protection must be ensured across distributed teams and cloud‐based infrastructures. At the same time, the computational footprint of large‐scale simulation and AI training must be co‐optimized with environmental and economic constraints, lest virtualization merely shift the burden of scaling from fabs to data centers.

In the long term, semiconductor engineering is likely to converge toward a regime in which **experimentation, qualification, and decision‐making are predominantly executed in virtual space**, with physical fabrication serving as periodic grounding rather than continuous iteration. In this regime, progress scales with the fidelity, integration, and governance of virtual evidence—not with wafer count or qualification cycle length. Virtualization thus emerges not as a temporary workaround for the end of geometric scaling, but as a durable organizing principle for how semiconductor innovation can continue in an era defined by complexity, constraint, and sustainability.

## Conflicts of Interest

The authors declare no conflicts of interest.

## Data Availability

The authors have nothing to report.

## References

[1] T. N. Theis and H.‐S. P. Wong , “The End of Moore's Law: A New Beginning for Information Technology,” Computing in Science & Engineering 19, no. 2 (2017): 41–50, 10.1109/mcse.2017.29.

[2] V. Moroz , X.‐W. Lin , P. Asenovet , et al., “DTCO Launches Moore's Law over the Feature Scaling Wall,” in 2020 IEEE International Electron Devices Meeting (IEDM) (IEEE, 2020), 41.1.1–41.1.4, 10.1109/iedm13553.2020.9372010.

[3] M. Waldrop , “More then Moore,” Nature 530, no. 11 (2016): 145, 10.1038/530144a.

[4] R. R. Schaller , “Moore's Law: Past, Present and Future,” IEEE Spectrum 34, no. 6 (1997): 52–59, 10.1109/6.591665.

[5] C. A. MacK , “Fifty Years of Moore's Law,” IEEE Transactions on Semiconductor Manufacturing 24, no. 2 (2011): 202–207, 10.1109/tsm.2010.2096437.

[6] M. Stettler , S. Cea , S. Hasan , et al., “State‐Of‐The‐Art TCAD: 25 Years Ago and Today,” in 2019 IEEE International Electron Devices Meeting (IEDM) (IEEE, 2019), 39.1.1–39.1.4, 10.1109/iedm19573.2019.8993451.

[7] R. H. Dennard , F. H. Gaensslen , H.‐N. Yu , V. L. Rideout , E. Bassous , and A. R. LeBlanc , “Design of Ion‐Implanted MOSFET's with Very Small Physical Dimensions,” IEEE Journal of Solid‐State Circuits 9, no. 5 (1974): 256–268, 10.1109/jssc.1974.1050511.

[8] S. Mukesh and J. Zhang , “A Review of the Gate‐All‐Around Nanosheet FET Process Opportunities,” Electronics 11, no. 21 (2022): 3589, 10.3390/electronics11213589.

[9] H. Guo , Z. Hu , Z. Liu , and J. Tian , “Stacking of 2D Materials,” Advanced Functional Materials 31, no. 4 (2021): 2007810, 10.1002/adfm.202007810.

[10] J. Tang , J. Jiang , X. Gao , et al., “Low‐Power 2D Gate‐All‐Around Logics via Epitaxial Monolithic 3D Integration,” Nature Materials 24, no. 4 (2025): 519–526, 10.1038/s41563-025-02117-w. 39953245

[11] K. E. Chong and K. C. Ng , “Relationship Between Overall Equipment Effectiveness, Throughput and Production Part Cost in Semiconductor Manufacturing Industry,” in 2016 IEEE International Conference on Industrial Engineering and Engineering Management (IEEM) (IEEE, 2024), 75–79, 10.1109/ieem.2016.7797839.

[12] J. Park , S. Y. Baek , J. Kim , S. J. Park , C. Lee , and C. Choi , “Practical Approaches on Cost Saving Strategies for Sustainable Semiconductor Manufacturing,” International Journal of Precision Engineering and Manufacturing‐Green Technology 12, no. 5 (2025): 1541–1557, 10.1007/s40684-024-00685-x.

[13] G. Refai‐Ahmed , V. V. Zhirnov , S. B. Park , et al., “New Roadmap for Microelectronics: Charting the Semiconductor Industry's Path over the Next 5, 10, and 20 Years,” in 2024 IEEE 26th Electronics Packaging Technology Conference (EPTC) (IEEE, 2024), 1260–1266, 10.1109/eptc62800.2024.10909778.

[14] Q. Zhang , Y. Hu , J. Yan , et al., “Large‐Language‐Model‐Based AI Agent for Organic Semiconductor Device Research,” Advanced Materials 36, no. 32 (2024): 2405163, 10.1002/adma.202405163. 38816034

[15] Z. Wang , F. Wang , L. Li , et al., “Quantum Kernel Learning for Small Dataset Modeling in Semiconductor Fabrication: Application to Ohmic Contact,” Advancement of Science 12, no. 35 (2025): 06213, 10.1002/advs.202506213. PMC12462921 40548955

[16] Z. Wang , S. Liu , K. Tao , et al., “Interpretable Surrogate Learning for Electronic Material Generation,” ACS Nano 18, no. 49 (2024): 33587–33601, 10.1021/acsnano.4c12166. 39485345

[17] C. Xin , Y. Yin , B. Song , Z. Fan , Y. Song , and F. Pan , “Machine Learning‐Accelerated Discovery of Novel 2D Ferromagnetic Materials with Strong Magnetization,” Chip 2, no. 4 (2023): 100071, 10.1016/j.chip.2023.100071.

[18] C. Jeong , S. Myung , B. Choi , et al., “Deep Learning for Semiconductor Materials and Devices Design,” in the 2023 7th IEEE Electron Devices Technology & Manufacturing Conference (EDTM) (IEEE, 2024), 1–3, 10.1109/edtm55494.2023.10103098.

[19] Y. Kwon and Y. Shin , “Calibration of Compact Resist Model Through CNN Training,” IEEE Transactions on Semiconductor Manufacturing 36, no. 2 (2023): 180–187, 10.1109/tsm.2023.3267670.

[20] P. Jungmann , J. B. Johnson , E. C. Silva , W. Taylor , A. H. Khan , and A. Kumar , “TCAD‐Enabled Machine Learning—An Efficient Framework to Build Highly Accurate and Reliable Models for Semiconductor Technology Development and Fabrication,” IEEE Transactions on Semiconductor Manufacturing 36, no. 2 (2023): 268–278, 10.1109/tsm.2023.3240033.

[21] K. Shiba , N. Miyashita , Y. Okada , and T. Sogabe , “Inverse Design of Intermediate Band Solar Cell via a Joint Drift‐Diffusion Simulator and Deep Reinforcement Learning Scheme,” Japanese Journal of Applied Physics 62, no. SK (2023): SK1046, 10.35848/1347-4065/acd34f.

[22] H. Wang , K. Wang , J.‐C. Wu , et al., “Inverse Design of InAs‐Based Interband Cascade Laser Active Regions via LightGBM‐Differential Evolution Co‐Optimization,” Journal of Physics D: Applied Physics 59, no. 2 (2026): 025116, 10.1088/1361-6463/ae30ef.

[23] T. S. Rawat , C.‐L. Hung , Y.‐K. Hsiao , et al., “A Reinforcement‐Learning Based Approach for Designing High‐Voltage SiC MOSFET Guard Rings,” IEEE Open Journal of Power Electronics 5 (2024): 1853–1861, 10.1109/ojpel.2024.3496865.

[24] Y. Xie , B. Davaji , I. Chakarov , et al., “Quantitative Comparison of Simulation and Experiment Enabling a Lithography Digital Twin,” IEEE Transactions on Semiconductor Manufacturing 37, no. 4 (2024): 546–552, 10.1109/tsm.2024.3427409.

[25] K. Kikuchi , M. Takada , W. Xueting , T. Ito , Y. Oomuro , and G. Li , “Quick Update of Virtual Metrology Using Weighted Transfer Lasso,” in 2024 International Symposium on Semiconductor Manufacturing (ISSM) , (IEEE, 2024), 1–4, 10.1109/issm64832.2024.10874907.

[26] E. Pitz , L. Medina , and M. Chopra , “Accelerating Chamber‐To‐Chamber Matching Using AI,” in 2024 35th Annual SEMI Advanced Semiconductor Manufacturing Conference (ASMC) , (IEEE, 2024), 1–5, 10.1109/asmc61125.2024.10545362.

[27] J. Kim , Y. Nam , M.‐C. Kang , et al., “Adversarial Defect Detection in Semiconductor Manufacturing Process,” IEEE Transactions on Semiconductor Manufacturing 34, no. 3 (2021): 365–371, 10.1109/tsm.2021.3089869.

[28] J. M. Gregoire , L. Zhou , and J. A. Haber , “Combinatorial Synthesis for AI‐Driven Materials Discovery,” Nature Synthesis 2, no. 6 (2023): 493–504, 10.1038/s44160-023-00251-4.

[29] F. Tsimpourlas , L. Papadopoulos , A. Bartsokas , and D. Soudris , “A Design Space Exploration Framework for Convolutional Neural Networks Implemented on Edge Devices,” IEEE Transactions on Computer‐Aided Design of Integrated Circuits and Systems 37, no. 11 (2018): 2212–2221, 10.1109/tcad.2018.2857280.

[30] D. Ivanov , A. Chezhegov , M. Kiselev , A. Grunin , and D. Larionov , “Neuromorphic Artificial Intelligence Systems,” Frontiers in Neuroscience 16 (2022): 959626, 10.3389/fnins.2022.959626. 36188479 PMC9516108

[31] Z. Wang , L. Li , R. C. C. Leon , et al., “Improving Semiconductor Device Modeling for Electronic Design Automation by Machine Learning Techniques,” IEEE Transactions on Electron Devices 71, no. 1 (2024): 263–271, 10.1109/ted.2023.3307051.

[32] Z. Wang , L. Li , and Y. Yao , “A Machine Learning‐Assisted Model for GaN Ohmic Contacts Regarding the Fabrication Processes,” IEEE Transactions on Electron Devices 68, no. 5 (2021): 2212–2219, 10.1109/ted.2021.3063213.

[33] K. Mehta , S. S. Raju , M. Xiao , B. Wang , Y. Zhang , and H. Y. Wong , “Improvement of TCAD Augmented Machine Learning Using Autoencoder for Semiconductor Variation Identification and Inverse Design,” IEEE Access 8 (2020): 143519–143529, 10.1109/access.2020.3014470.

[34] C. Jeong , S. Myung , I. Huh , et al., “Bridging TCAD and AI: Its Application to Semiconductor Design,” IEEE Transactions on Electron Devices 68, no. 11 (2021): 5364–5371, 10.1109/ted.2021.3093844.

[35] N. Wang , W. Yan , Y. Qu , S. Ma , S. Z. Li , and M. Qiu , “Intelligent Designs in Nanophotonics: From Optimization Towards Inverse Creation,” PhotoniX 2, no. 1 (2021): 22, 10.1186/s43074-021-00044-y.

[36] Q. Wang , M. Makarenko , A. Burguete Lopez , F. Getman , and A. Fratalocchi , “Advancing Statistical Learning and Artificial Intelligence in Nanophotonics Inverse Design,” Nanophotonics 11, no. 11 (2022): 2483–2505, 10.1515/nanoph-2021-0660. 39635678 PMC11502023

[37] S. Lee , M. Sim , Y. Kang , D. Kim , and H.‐S. Lee , “Bayesian‐Optimization‐Based Approach for Sheet‐Resistance Control in Silicon Wafers Toward Automated Solar‐Cell Manufacturing,” Materials Science in Semiconductor Processing 198 (2025): 109759, 10.1016/j.mssp.2025.109759.

[38] H. Jeong , J. Choi , H. Cho , et al., “MOBO‐Driven Advanced Sub‐3‐nm Device Optimization for Enhanced PDP Performance,” IEEE Transactions on Electron Devices 71, no. 5 (2024): 2881–2887, 10.1109/ted.2024.3378224.

[39] J. Chen , H. Li , H. Tan , et al., “Intelligent Design of Superjunction Devices Based on Physics‐Informed Neural Network,” in 2025 37th International Symposium on Power Semiconductor Devices and ICs (ISPSD) (IEEE, 2025), 449–452, 10.23919/ispsd62843.2025.11118128.

[40] H. Jeong , J. Choi , Y. Kim , J.‐T. Kong , and S. Kim , “Efficient Neural Network‐Based Compact Modeling for Novel Device Structures Using a Multi‐Fidelity Model and Active Learning,” Electronics 13, no. 23 (2024): 4840, 10.3390/electronics13234840.

[41] S. Mondal , S. Ray , A. Acharyya , et al., “Accelerated Prediction of Terahertz Performance Metrics in GaN IMPATT Sources via Artificial Neural Networks,” IEEE Access 13 (2025): 84284–84302, 10.1109/access.2025.3567410.

[42] P. Raut , D. K. Panda , and A. K. Goyal , “A Comprehensive Review on Next‐Generation Modeling and Optimization for Semiconductor Devices,” IEEE Access 13 (2025): 123724–123742, 10.1109/access.2025.3587721.

[43] S. Mishra , B. Gaikwad , and N. Chaturvedi , “Prediction of Threshold Voltage of GaN HEMTs Using Deep Learning Model Designed by Genetic Algorithm,” Materials Science in Semiconductor Processing 152 (2022): 107057, 10.1016/j.mssp.2022.107057.

[44] S. Kim , K. Lee , H.‐K. Noh , et al., “Automatic Modeling of Logic Device Performance Based on Machine Learning and Explainable AI,” in 2020 International Conference on Simulation of Semiconductor Processes and Devices (SISPAD) (IEEE, 2020), 47–50, 10.23919/sispad49475.2020.9241681.

[45] W. T. Osowiecki , M. J. Coogans , S. Sriraman , R. Ranjan , Y. J. Lu , and D. M. Fried , “Achieving Sustainability in the Semiconductor Industry: the Impact of Simulation and AI,” IEEE Transactions on Semiconductor Manufacturing 37, no. 4 (2024): 464–474, 10.1109/tsm.2024.3438622.

[46] G. Fan , T. Ma , X. Sun , L. Shao , and K. L. Low , “Graph Attention Network‐Based Unified TCAD Modeling Enabling Fast Design Technology Co‐Optimization through Transfer Learning,” IEEE Transactions on Electron Devices 72, no. 1 (2025): 474–481, 10.1109/ted.2024.3493854.

[47] A. J. García‐Loureiro , N. Seoane , J. G. Fernández , and E. Comesaña , “A General Toolkit for Advanced Semiconductor Transistors: From Simulation to Machine Learning,” IEEE Journal of the Electron Devices Society 12 (2024): 1057–1064, 10.1109/jeds.2024.3401852.

[48] S.‐C. Han , J. Choi , and S.‐M. Hong , “Acceleration of Three‐Dimensional Device Simulation with the 3D Convolutional Neural Network,” in 2021 International Conference on Simulation of Semiconductor Processes and Devices (SISPAD) (IEEE, 2021), 52–55, 10.1109/sispad54002.2021.9592540.

[49] A. Gu , M. Terada , H. Stegmann , T. Rodgers , C. Fu , and Y. Yang , “From System to Package to Interconnect: An Artificial Intelligence Powered 3D X‐Ray Imaging Solution for Semiconductor Package Structural Analysis and Correlative Microscopic Failure Analysis,” in 2022 IEEE International Symposium on the Physical and Failure Analysis of Integrated Circuits (IPFA) (IEEE, 2022), 1–5, 10.1109/ipfa55383.2022.9915756.

[50] J. Shi , C. Chen , X. Li , H. Chen , H. Lin , and Z. Chen , “Optoelectronic Synaptic Transistors via Adding Insulator into Semiconductor for Brain‐Inspired Computing,” IEEE Transactions on Electron Devices 71, no. 11 (2024): 6989–6995, 10.1109/ted.2024.3454033.

[51] Y.‐P. Chen , V. Karkaria , Y.‐K. Tsai , et al., “Real‐Time Decision‐Making for Digital Twin in Additive Manufacturing with Model Predictive Control Using Time‐Series Deep Neural Networks,” Journal of Manufacturing Systems 80 (2025): 412–424, 10.1016/j.jmsy.2025.03.009.

[52] A. H. Sakr , A. Aboelhassan , S. Yacout , and S. Bassetto , “Simulation and Deep Reinforcement Learning for Adaptive Dispatching in Semiconductor Manufacturing Systems,” Journal of Intelligent Manufacturing 34, no. 3 (2023): 1311–1324, 10.1007/s10845-021-01851-7.

[53] I.‐B. Park , J. Huh , J. Kim , and J. Park , “A Reinforcement Learning Approach to Robust Scheduling of Semiconductor Manufacturing Facilities,” IEEE Transactions on Automation Science and Engineering 17, no. 3 (2020): 1420–1431, 10.1109/tase.2019.2956762.

[54] S. Behrendt , T. Altenmüller , M. C. May , A. Kuhnle , and G. Lanza , “Real‐To‐Sim: Automatic Simulation Model Generation for a Digital Twin in Semiconductor Manufacturing,” Journal of Intelligent Manufacturing 37, no. 2 (2025): 829–848, 10.1007/s10845-025-02572-x.

[55] J. Hwang and S. D. Noh , “Digital Twin‐Based Optimization of Operational Parameters for Cluster Tools in Semiconductor Manufacturing,” IEEE Access 12 (2024): 122078–122100, 10.1109/access.2024.3450869.

[56] T. Tsutsui and T. Matsuzawa , “Virtual Metrology Model Robustness Against Chamber Condition Variation Using Deep Learning,” IEEE Transactions on Semiconductor Manufacturing 32, no. 4 (2019): 428–433, 10.1109/tsm.2019.2931328.

[57] S. Kim , S. Zhang , and Y. Shin , “ML‐Guided Curvilinear OPC: Fast, Accurate, and Manufacturable Curve Correction,” IEEE Transactions on Semiconductor Manufacturing 38, no. 1 (2025): 19–28, 10.1109/tsm.2025.3527514.

[58] X. Liang , Y. Ouyang , H. Yang , B. Yu , and Y. Ma , “RL‐OPC: Mask Optimization with Deep Reinforcement Learning,” IEEE Transactions on Computer‐Aided Design of Integrated Circuits and Systems 43, no. 1 (2024): 340–351, 10.1109/tcad.2023.3309745.

[59] M. Liu , Z. Tang , H. You , et al., “An Efficient Machine Learning‐Enhanced DTCO Framework for Low‐Power and High‐Performance Circuit Design,” Journal of Information and Intelligence 3, no. 3 (2025): 194–209, 10.1016/j.jiixd.2025.02.001.

[60] C.‐K. Cheng , C.‐T. Ho , C. Holtz , D. Lee , and B. Lin , “Machine Learning Prediction for Design and System Technology Co‐Optimization Sensitivity Analysis,” IEEE Transactions on Very Large Scale Integration (VLSI) Systems 30, no. 8 (2022): 1059–1072, 10.1109/tvlsi.2022.3172938.

[61] M. Kouda , Y. Mori , T. Sugita , and Y. Ng , “Optimal Design of Wet Etching Bath for 3D Flash Memories Using Multi‐Objective Bayesian Optimization,” IEEE Transactions on Semiconductor Manufacturing 38, no. 3 (2025): 439–445, 10.1109/tsm.2025.3569278.

[62] H.‐K. Wang , Y.‐C. Lin , T.‐Y. Chen , C.‐C. Wang , and C.‐P. Hung , “Optimizing Warpage in Semiconductor Packaging Using GA‐NN and Conditional GAN with Explainable AI,” Computers & Industrial Engineering 206 (2025): 111246, 10.1016/j.cie.2025.111246.

[63] C. Bunne , Y. Roohani , Y. Rosen , et al., “How to Build the Virtual Cell with Artificial Intelligence: Priorities and Opportunities,” Cell 187, no. 25 (2024): 7045–7063, 10.1016/j.cell.2024.11.015. 39672099 PMC12148494

[64] M. P. McLaughlin , P. Mennell , A. Stamper , et al., “Improved Color Defect Detection with Machine Learning for after Develop Inspections in Lithography,” IEEE Transactions on Semiconductor Manufacturing 35, no. 3 (2022): 418–424, 10.1109/tsm.2022.3186607.

[65] G. Cho , Y. Kwon , P. Kareem , and Y. Shin , “Integrated Test Pattern Extraction and Generation for Accurate Lithography Modeling,” IEEE Transactions on Semiconductor Manufacturing 35, no. 3 (2022): 495–503, 10.1109/tsm.2022.3184412.

[66] H. E. Sim , M. U. Lee , and S. J. Hong , “Virtual Metrology of Multiple Dielectric Layer Thickness for 3D‐NAND Deposition Process,” IEEE Transactions on Semiconductor Manufacturing 38, no. 2 (2025): 240–250, 10.1109/tsm.2025.3537974.

[67] H. Stegmann and F. Cognigni , “Few‐Shot AI Segmentation of Semiconductor Device FIB‐SEM Tomography Data,” Journal of Failure Analysis and Prevention 25 (2025): 2055–2069, 10.1007/s11668-025-02203-w.

[68] D. Kim , W. Lee , Y. Yim , et al., “Interactive Image Annotation and AI‐Assisted Segmentation of TEM Images for Automatic CD Measurement,” in Metrology, Inspection, and Process Control XXXVIII (SPIE, 2024), 231–240, 10.1117/12.3009407.

[69] Y. Yang and M. Sun , “Knowledge Distillation Cross Domain Diffusion Model: A Generative AI Approach for Defect Pattern Segmentation,” IEEE Transactions on Semiconductor Manufacturing 37, no. 4 (2024): 634–642, 10.1109/tsm.2024.3472611.

[70] T. Patel , R. Murugan , G. Yenduri , R. H. Jhaveri , H. Snoussi , and T. Gaber , “Demystifying Defects: Federated Learning and Explainable AI for Semiconductor Fault Detection,” IEEE Access Practical Innovation Open Solution 12 (2024): 116987–117007, 10.1109/access.2024.3425226.

[71] Y. Fassi , V. Heiries , J. Boutet , and S. Boisseau , “Physics‐Informed Machine Learning for Robust Remaining Useful Life Estimation of Power MOSFETs,” in 2024 IEEE International Conference on Prognostics and Health Management (ICPHM) (IEEE, 2024), 399–406, 10.1109/icphm61352.2024.10626501.

[72] J. Park , J. Bae , J. Lim , B. Kim , and J. Jeong , “LED‐Display Defect Detection Based on YOLOv5 and Transformer,” IEEE Access Practical Innovation Open Solution 11 (2023): 124660–124675, 10.1109/access.2023.3325487.

[73] O. Kwon , N. Lee , and K. Kim , “Improvement of Virtual Diagnostics Performance for Plasma Density in Semiconductor Etch Equipment Using Variational Auto‐Encoder,” IEEE Transactions on Semiconductor Manufacturing 35, no. 2 (2022): 256–265, 10.1109/tsm.2022.3154366.

[74] L. Xin , X. Liu , Z. Yang , X. Zhang , Z. Gao , and Z. Liu , “Three‐Dimensional Reconstruction of Super‐Resolved White‐Light Interferograms Based on Deep Learning,” Optics and Lasers in Engineering 145 (2021): 106663, 10.1016/j.optlaseng.2021.106663.

[75] X. Qi , Y. Lian , Y. Wang , and Z. Lu , “Simulation‐Driven End‐to‐End Deep Learning Method for White‐Light Interference Topography Reconstruction,” Photonics 12, no. 7 (2025): 702, 10.3390/photonics12070702.

[76] H. Villarraga‐Gómez , K. Crosby , M. Terada , and M. N. Rad , “Assessing Electronics with Advanced 3D X‐Ray Imaging Techniques, Nanoscale Tomography, and Deep Learning,” Journal of Failure Analysis and Prevention 24, no. 5 (2024): 2113–2128, 10.1007/s11668-024-01989-5.

[77] J. T. Mathew , A. Inobeme , Y. Azeh , et al., “Total Reflection X‐Ray Fluorescence: Technological Developments and Expanding Applications,” in X‐Ray Fluorescence Spectroscopy and Chemometrics, (Springer Nature Switzerland, 2025), 303–323, 10.1007/978-3-031-98375-7_13.

[78] V. Yim , A. Mukhtarov , N. Drogue , et al., “TXRF Capability of Metallic Contamination Analysis on Rough Silicon Wafers,” Journal of Materials Research 39, no. 18 (2024): 2522–2530, 10.1557/s43578-024-01401-w.

[79] A. Jo and W. Lee , “Brass Material Analysis with Deep‐Learning‐Based CdTe Semiconductor X‐Ray Fluorescence System,” IEEE Transactions on Nuclear Science 69, no. 5 (2022): 1085–1091, 10.1109/tns.2022.3165318.

[80] S. Shan , F. Zhao , Z. Li , L. Luo , and X. Li , “A Comprehensive Review of Optical Metrology and Perception Technologies,” Sensors 25, no. 22 (2025): 6811, 10.3390/s25226811. 41305020 PMC12656061

[81] K. Grobe , “Simplified LCA for Lifetime‐Limitation Indication,” in 2024 Electronics Goes Green 2024+ (EGG) (IEEE, 2024), 1–6, 10.23919/egg62010.2024.10631240.

[82] D.‐Y. Wang , Y. L. Lin , Y. T. Chen , et al., “Artificial Intelligence‐Based Reliability and Material Characterization of AlInGaP LEDs in Salty Water Environments,” IEEE Access Practical Innovation Open Solution 12 (2024): 170936–170945, 10.1109/access.2024.3497600.

[83] J. Zhang , J. Wu , O. Stroyuk , et al., “Self‐Driving AMADAP Laboratory: Accelerating the Discovery and Optimization of Emerging Perovskite Photovoltaics,” MRS Bulletin 49, no. 12 (2024): 1284–1294, 10.1557/s43577-024-00816-4.

[84] J. Zhang , J. A. Hauch , and C. J. Brabec , “Toward Self‐Driven Autonomous Material and Device Acceleration Platforms (AMADAP) for Emerging Photovoltaics Technologies,” Accounts of Chemical Research 57, no. 9 (2024): 1434–1445, 10.1021/acs.accounts.4c00095. 38652511 PMC11079961

[85] S. Kikuta , M. Yamagami , H. Kono , and M. Doi , “Improvement of Sensitivity in Total Reflection X‐Ray Fluorescence Spectrometry by Machine Learning,” Bunseki Kagaku 69, no. 9 (2020): 463–470, 10.2116/bunsekikagaku.69.463.

[86] Y. Lee and S. B. Kim , “Weakly Supervised Image Segmentation for Detecting Defects From Scanning Electron Microscopy Images in Semiconductor,” IEEE Access 12 (2024): 184896–184908, 10.1109/access.2024.3513873.

[87] H. Yun , H. Jang , S. Lee , et al., “Nonlinear Variation Decomposition of Neural Networks for Holistic Semiconductor Process Monitoring,” Advanced Intelligent Systems 6, no. 10 (2024): 2300920, 10.1002/aisy.202300920.

[88] H.‐H. Hsiao and K.‐J. Wang , “HotspotFusion: a Generative AI Approach to Predicting CMP Hotspot in Semiconductor Manufacturing,” IEEE Transactions on Semiconductor Manufacturing 38, no. 1 (2025): 73–82, 10.1109/tsm.2024.3510376.

[89] J. Park , J. Lee , and J. Jeong , “LyFormer Based Object Detection in Reel Package X‐Ray Images of Semiconductor Component,” Journal of King Saud University—Computer and Information Sciences 36, no. 1 (2024): 101859, 10.1016/j.jksuci.2023.101859.

[90] X. Wu , S. Shi , J. Jiang , et al., “Bionic Olfactory Neuron with In‐Sensor Reservoir Computing for Intelligent Gas Recognition,” Advanced Materials 37, no. 13 (2025): 2419159, 10.1002/adma.202419159. 39945055

[91] N. Hari , M. Ahsan , S. Ramasamy , P. Sanjeevikumar , A. Albarbar , and F. Blaabjerg , “Gallium Nitride Power Electronic Devices Modeling Using Machine Learning,” IEEE Access 8 (2020): 119654–119667, 10.1109/access.2020.3005457.

[92] Y. Zheng , H. Xu , Z. Li , et al., “Artificial Intelligence‐Driven Approaches in Semiconductor Research,” Advanced Materials 37, no. 35 (2025): 2504378, 10.1002/adma.202504378. 40534303

[93] A. C. Huang , S. H. Meng , and T. J. Huang , “A Survey on Machine and Deep Learning in Semiconductor Industry: Methods, Opportunities, and Challenges,” Cluster Computing 26, no. 6 (2023): 3437–3472, 10.1007/s10586-023-04115-6.

[94] Y. Yin and Y. Yang , “Sustainable Transition of the Global Semiconductor Industry: Challenges, Strategies, and Future Directions,” Sustainability 17, no. 7 (2025): 3160, 10.3390/su17073160.

[95] K.‐H. L. Loh , “Enabling Generative AI: Innovations and Challenges in Semiconductor Design Technologies,” in 2025 Symposium on VLSI Technology and Circuits (VLSI Technology and Circuits) (IEEE, 2025), 1–6, 10.23919/vlsitechnologyandcir65189.2025.11074926.

[96] D.‐Y. Son , J.‐H. Lee , and C. Oh , “A Study of the Technological Development Direction and Economic Security of the AI Semiconductor Industry: with Emphasis on an Analysis Using U.S. Patent Data,” Journal of Digital Contents Society 24, no. 7 (2023): 1555–1565, 10.9728/dcs.2023.24.7.1555.

[97] Z. Wang , T. Van Der Laan , and M. Usman , “Self‐Adaptive Quantum Kernel Principal Component Analysis for Compact Readout of Chemiresistive Sensor Arrays,” Advanced Science 12 (2025): 2411573, 10.1002/advs.202411573. 39854057 PMC12005759

